# Aligning neonatal sepsis triage diagnostics with WHO target product profiles in low- and middle-income countries

**DOI:** 10.1136/bmjgh-2025-023363

**Published:** 2026-06-16

**Authors:** Birgitta Gleeson, Benjamin Blumel, Ana Belen Ibarz Pavon, Rebecca P Kirby, Cannon Hansen, Berra Erkosar, Naomi E Spotswood, Yasir Bin Nisar, Kara M Palamountain

**Affiliations:** 1 AMR, FIND, Geneva, Switzerland; 2 FIND, Geneva, Switzerland; 3 Northwestern University Kellogg School of Management, Evanston, Illinois, USA; 4 Department of Bioengineering, Rice University, Houston, Texas, USA; 5 Burnet Institute, Melbourne, Victoria, Australia; 6 Faculty of Medicine, Dentistry and Health Sciences, University of Melbourne, Melbourne, Victoria, Australia; 7 Department of Maternal, Newborn, Child and Adolescent Health and Ageing, World Health Organization, Geneve, Switzerland

**Keywords:** Global Health, Review, Diagnostics and tools

## Abstract

Neonatal sepsis is a leading cause of infant morbidity and mortality in low- and middle-income countries (LMICs), yet despite this, progress in the development and implementation of effective diagnostics has been limited. Current diagnostic methods rely on clinical assessments of nonspecific signs and symptoms, limited laboratory testing and technologies that are inaccessible, unaffordable or unsuitable for resource-constrained settings. There is an urgent need for reliable, affordable and context-appropriate point-of-care diagnostics.

This analysis synthesises evidence across current and emerging diagnostic strategies for neonatal sepsis, including clinical algorithms, host-response biomarkers, haematological parameters, continuous monitoring and artificial intelligence-based digital platforms. We assess their performance, operational feasibility in LMICs and alignment with the WHO Target Product Profile (TPP) for neonatal sepsis, including requirements for highly sensitive triage, feasibility in non-hospital settings and suitability for use at the point of care in LMICs.

We identified promising existing technologies and emerging approaches that could be used to address the challenges of diagnosing neonatal sepsis in LMICs. These include host-response biomarkers that offer greater accuracy than those currently used, as well as affordable technologies that can improve current predictive algorithms. Taken together, these findings support a move towards integrated, TPP-aligned diagnostic strategies that combine affordable point-of-care testing, routine clinical data and digital decision support to improve early detection, rationalise antibiotic use and reduce neonatal mortality.

**Summary box**

- Despite the extremely high disease burden and mortality associated with neonatal sepsis in low- and middle-income countries, progress in developing diagnostics for neonatal sepsis remains slow.
- Nonspecific clinical signs and symptoms make neonatal sepsis challenging to diagnose, which leads to overtreatment with empirical antibiotics and delayed treatment of true infections, contributing to poor outcomes and antimicrobial resistance.
- The recently published WHO Target Product Profile provides a framework for the performance and operational requirements of neonatal sepsis diagnostics.
- Emerging technologies, including lower-cost point-of-care diagnostics, continuous monitoring and artificial intelligence, have made the development of timely and accurate diagnostics for neonatal sepsis more feasible.
- A health system integrated, artificial intelligence-enabled triage approach combining physiological monitoring, laboratory parameters and host-response biomarkers has the potential to align with WHO requirements and improve clinical decision-making in low- and middle-income countries.

## Introduction

 Neonatal sepsis is the leading cause of infant morbidity and mortality worldwide, with the highest burden in low- and middle-income countries (LMICs), yet the availability of diagnostic tools in these countries remains limited.^1 2^ Clinically suspected sepsis is estimated to affect 166 per 1000 live births in LMICs, with an all-cause mortality rate of 0.83 per 1000 neonate-days.^3^ Gram-negative bacteria, which are intrinsically difficult to treat, are now the predominant cause of neonatal sepsis in LMICs, blurring the traditional distinction between early- and late-onset sepsis.^4 5^ The complexity of the neonatal immune response has made the development of diagnostic tools challenging. This is further compounded by a lack of global consensus on how to define neonatal sepsis. A 2021 review identified 128 unique definitions, reflecting the heterogeneity of diagnostic approaches and underscoring the need for consensus to facilitate comparisons across scientific evidence.^6^ These challenges are particularly acute in LMICs, where limited diagnostic capacity, differing pathogens, unknown resistance profiles and infrastructural constraints compel healthcare workers to rely on the use of broad-spectrum empirical antibiotic therapy.^7^ Blood culture remains the gold standard for diagnosing neonatal sepsis and confirming the presence of a microorganism. However, its limitations, including slow processing times and low sensitivity because of large volume requirements, preclude its use for the early identification of sepsis.^8^,^10^ While early detection and prompt intervention with appropriate antimicrobial treatment is essential to improve survival rates,^11^ empirical treatment poses risks, including prolonged hospitalisation, treatment-related adverse events and increased mortality.^12 13^

Inappropriate antibiotic use coupled with high levels of antibiotic resistance is associated with significantly more neonatal sepsis deaths in LMICs.^14 15^ Critically, as the global prevalence of antimicrobial resistance (AMR) is on the rise, the United Nations High-Level Meeting on AMR in 2024 reinforced a focus on medical countermeasures, including diagnostics, as a tool to combat AMR. The WHO has published a target product profile (TPP) highlighting the need for diagnostic innovation for neonatal sepsis.^11^ The test described in the TPP is not intended for universal screening at birth but for use in newborns or young infants in the diagnostic ‘grey zone’, where the pretest probability of infection is higher. This includes infants who are not well enough to be discharged and not obviously critically unwell, but who have non-specific or mild signs (eg, poor feeding, lethargy, temperature instability, mild breathing difficulty) or risk factors for infection (eg, preterm, low birth weight, maternal infection). In non-hospital settings, where resources are limited, it should be used for referral to higher levels of care and/or for initiating antibiotics, and in hospital settings for initiating sepsis management and antibiotics. Advances in biomarker discovery, novel technologies and artificial intelligence (AI) offer opportunities to meet these requirments. In this analysis, we evaluate existing and emerging diagnostic approaches, ranging from clinical algorithms to novel biomarkers and wearable sensors, and propose an integrated, AI-enabled diagnostic framework that could address the constraints of LMIC health systems.

### Clinical assessment and algorithms

In the absence of a single test that provides diagnostic certainty, models combining multiple clinical and laboratory parameters have been used in clinical practice to enhance the accuracy of diagnosing neonatal sepsis. A scoping review of 44 distinct models revealed wide variability in their performance in LMICs, indicating that no single approach consistently meets WHO TPP thresholds.^16^ This variability is likely due to the lack of a standardised methodological approach to assessing the performance of clinical models, including the use of various reference standards such as blood culture, clinical assessment, or a combination of these and varied study populations. The Kaiser Permanente (KP) neonatal sepsis risk score incorporates maternal risk factors and clinical factors without requiring laboratory tests. This score is widely used in hospitals across the USA and other high income countries^17 18^ for the detection of early-onset sepsis, but it has only been assessed in a few studies in LMICs. These limited evaluations report widely variable performance, with median sensitivities and specificities of 81.2% (range 54.6%–85.7%) and 87.8% (range 44.3%–93.9%), respectively.^16^ The widespread use of the KP score has been questioned due to the risk of missed diagnoses, especially given its wide performance range in different settings.^19^ The haematological scoring system reported in the same scoping review demonstrated slightly higher median sensitivity and specificity across 24 studies, at 84.4% (range 10.5%–100%) and 87.5% (22.5%–100%), respectively.^16^ Despite the considerable variability, this score uses routine blood analysis, including differential white blood cell (WBC) counts and blood smears, making it relatively suitable for use in hospitals with laboratory capacity in LMICs. Several other scores tend to perform more poorly in LMIC contexts, as confirmed by a South African study that evaluated five of eleven prediction scores identified for their suitability for use in LMICs. Five scores (NOSEP1, NOSEP1-NEW1, Singh, Rosenberg and Bekhof)^20^,^24^ were retrospectively evaluated in a cohort of infants with very low birth weights, with sensitivities ranging from 3% (NOSEP1) to 74% (Singh) and specificities of 31%–100% when assessed against both culture-confirmed and clinically presumed neonatal sepsis.^22^ All models performed worse than in their original validation settings, which included Europe and South East Asia, underscoring the need for validation in specific settings prior to clinical use in LMIC settings.^22^

Further illustrating the challenge of generalisability, two other prediction models developed and assessed in Thailand also present practical obstacles to their widespread use in LMIC settings. The Okascharoen score, derived from data from a university hospital in Bangkok, reported a 97% sensitivity and 70% negative predictive value for late-onset sepsis,^25^ but it is dependent on neonatal blood-pressure monitoring, which may not be widely available in LMICs. The Queen Sirikit score demonstrated 88.5% sensitivity and 90.4% specificity in proven sepsis cases, including positive bacterial culture results, PCR, gram-staining, latex agglutination tests/antigen-antibody detection for bacteria.^26^ This score requires measuring pH, which may be challenging to perform routinely neonatal units lacking access to blood-gas analysers.

Recognising that a considerable number of neonatal sepsis cases are identified outside of hospital settings, the WHO TPP also prioritises the diagnosis of neonatal sepsis in a non-hospital setting.^11^ Several studies have investigated the use of various clinical signs to aid in diagnosing neonatal sepsis in these settings. The Young Infants Clinical Signs Study, an extensive, multi-country study conducted in Bangladesh, Bolivia, Ghana, India, Pakistan and South Africa, identified 12 signs or symptoms that could be used to predict severe illness in the first week of life among infants who were brought to health facilities.^27^ The presence of any one of these signs or symptoms predicted severe illness with a sensitivity and specificity of 87% and 74%, respectively, when assessed against an expert paediatrician who determined the presence of severe illness that required hospital admission. WHO subsequently used these findings to inform the guidelines for the management of serious bacterial infection and updated the Integrated Management of Childhood Illness protocols.^28 29^ In another community-based study conducted in the Gadchiroli district of India, village health workers trained in neonatal care used a specific set of symptoms to diagnose neonatal sepsis during home-based neonatal care. During the intervention’s first 3 years, the case fatality in neonatal sepsis declined from 16.6% before treatment to 2.8% after treatment by village health workers.^30^ During the 7-year study period, village health workers correctly identified 89% of neonatal sepsis cases, resulting in a nearly 60% reduction in case fatality. While this improvement could not be attributed solely to timely and accurate diagnosis, the study demonstrated that home-based management of neonates by carefully selected and appropriately trained and supervised healthcare workers may be beneficial in LMICs, where access to hospital care is often limited.^31^ Notably, during the study’s non-intervention period, a simple rule requiring the presence of just two of seven clinical signs was found to predict sepsis-related mortality with 100% sensitivity and 92% specificity, although these findings should be interpreted cautiously because of limited generalisability.^32^

The ideal prediction model should rely on simple and readily available clinical and/or laboratory tests.^22 26^ The most frequently used predictors are WBC counts, C reactive protein (CRP) level, platelet counts and neonatal fever, as well as gestational age and neutrophil counts.^16^ While existing models have performances lower than the WHO TPP requirements, it is now more feasible than ever to enhance the performance of these predictive models. Novel host-response biomarkers, advanced cell-imaging techniques, affordable point-of-care (POC) technologies and machine learning/AI-based technologies will aid in the development of more sophisticated and accurate prediction tools. Critically, these models must be validated within the specific context for which they are intended. To date, such validation has been insufficient in low resource settings.^16 33^

### Biomarkers for sepsis triage

**Figure 1:**
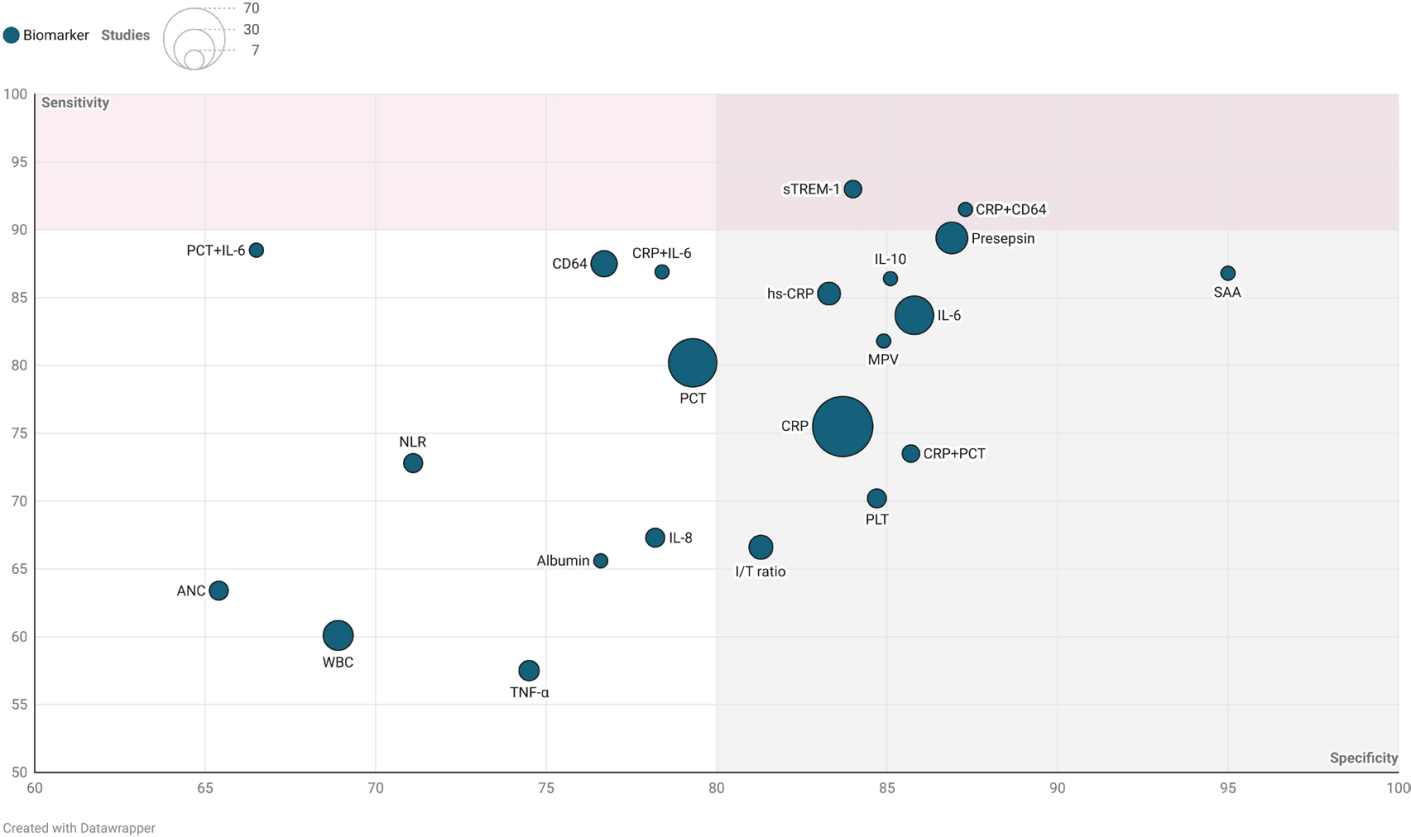
Pooled specificity and sensitivity estimates for biomarkers included in the analysis. Data were obtained from 113 studies identified through a PubMed-based rapid evidence synthesis. Pooled estimates were generated to illustrate comparative biomarker performance. As per the WHO TPP, the shaded regions correspond to the essential performance criteria (sensitivity ≥90% and specificity ≥80%). ANC, absolute neutrophil count; CRP, C reactive protein; hs-CRP, high sensitivity CRP; IL, interleukin; I/T ratio, immature-to-total neutrophil ratio; MPV, mean platelet volume; NLR, neutrophil-to-lymphocyte ratio; PCT, procalcitonin; PI, prediction interval; PLT, platelet count; SAA, serum amyloid A; sTREM-1, soluble triggering receptor expressed on myeloid cells-1; TNFα, tumour necrosis factor alpha; TPP, target product profile; WBC, white blood cell.

Host-response biomarkers can enhance the triage and diagnosis of neonatal sepsis. According to the WHO TPP, an ideal test should demonstrate high sensitivity (desirable: ≥95%, essential: ≥90%) and specificity (non-hospital test/hospital test— desirable: ≥90%, essential: ≥70%/80%) and should be measurable at the POC.^11 34^ To identify biomarkers that could meet these performance standards, we conducted a rapid synthesis of evidence from 113 studies, including 27 886 neonates (figure 1 and online supplemental table 1). To identify the performance of the biomarkers across the studies we used pooled estimates in our analysis (see online supplemental file 1 for additional Methods).

Our analysis identified that the single biomarkers with the greatest balanced accuracy were serum amyloid A (SAA) (90.9%), soluble triggering receptor expressed on myeloid cells-1 (sTREM-1) (88.5%), presepsin (88.2%) and interleukin (IL)-10 (85.8%) (table 1). The best-performing combinations were CRP+CD64 (89.4%) and CRP+IL-6 (82.65%). While CRP+CD64 demonstrated both high pooled sensitivity (91.5%) and specificity (87.3%), the small number of studies and wide 95% confidence and prediction intervals make the true specificity uncertain. Pooled sensitivity estimates showed that only sTREM-1 and CRP+CD64 met the minimum TPP requirements, although both had wide 95% confidence and prediction intervals, with lower bounds falling below the TPP’s 90% threshold. Notably, CRP+IL-6, although based on a limited number of studies, demonstrated performance estimates that were no better than those of IL-6 alone (sensitivity 86.9%, specificity 78.4%). While some biomarkers met the minimum TPP specificity criteria, the prediction intervals were generally wide, and only CRP+IL-6 had a lower bound above the TPP minimum of 70%.

**Table 1:** Summary of biomarkers included in the analysis, together with sensitivity, specificity and balanced accuracy estimates

Biomarker	Number of studies (datasets)	Sensitivity (95% CI) (95% PI)	Specificity (95% CI) (95% PI)	Balanced accuracy†
SAA	3	86.8 (71.0 to 94.6) (53.6 to 97.4)	95 (35.0 to 99.8) (2.1 to 100)	90.9
CRP+CD64	3	91.5 (72.7 to 97.7) (44.7 to 99.3)	87.3 (15.5 to 99.6) (0.6 to 100)	89.4
sTREM-1	5	93 (79.2 to 97.9) (46.1 to 99.5)	84 (60.6 to 94.7) (24.4 to 98.9)	88.5
Presepsin	18	89.4 (85.1 to 92.6) (70.9 to 96.7)	86.9 (81.5 to 90.9) (62.5 to 96.4)	88.15
IL-10	3	86.4 (82.3 to 89.6) (80.6 to 90.6)	85.1 (42.9 to 97.7) (11.2 to 99.6)	85.75
IL-6	27	83.7 (77.6 to 88.4) (45.9 to 96.9)	85.8 (79.8 to 90.3) (44.7 to 97.8)	84.75
hs-CRP	9	85.3 (74.1 to 92.1) (43.8 to 97.7)	83.3 (62.8 to 93.6) (17.1 to 99.2)	84.3
MPV	3	81.8 (44.9 to 96.10) (15.2 to 99.1)	84.9 (70.0 to 93.1) (51.7 to 96.7)	83.35
CRP+IL-6	3	86.9 (78.8 to 92.2) (72.0 to 94.4)	78.4 (72.9 to 83.0) (71.9 to 83.6)	82.65
CD64	12	87.5 (82.7 to 91.1) (72.8 to 94.8)	76.7 (65.9 to 84.8) (39.1 to 94.4)	82.1
PCT	43	80.2 (75.8 to 83.9) (48.8 to 94.5)	79.3 (74.9 to 83.1) (47.2 to 94.3)	79.75
CRP	67	75.5 (70.5 to 79.8) (32.1 to 95.2)	83.7 (79.7 to 87.0) (41.8 to 97.4)	79.6
CRP+PCT	5	73.5 (57.6 to 85.0) (33.9 to 93.8)	85.7 (69.1 to 94.1) (36.5 to 98.4)	79.6
PCT+IL-6	3	88.5 (81.6 to 93.0) (76.6 to 94.8)	66.5 (56.4 to 75.2) (49.9 to 79.8)	77.5
PLT	6	70.2 (51.3 to 84.1) (25.5 to 94.2)	84.7 (73.5 to 91.7) (52.6 to 96.5)	77.45
I/T ratio	10	66.6 (44.2 to 83.4) (9.9 to 97.3)	81.3 (63.5 to 91.6) (20.8 to 98.6)	73.95
IL-8	6	67.3 (50.5 to 80.7) (27.3 to 91.9)	78.2 (54.7 to 91.5) (18.1 to 98.3)	72.75
NLR	6	72.8 (61.5 to 81.8) (43.3 to 90.4)	71.1 (52.7 to 84.4) (25.6 to 94.6)	71.95
Albumin	3	65.6 (40.5 to 84.3) (20.0 to 93.6)	76.6 (61.1 to 87.2) (44.0 to 93.1)	71.1
TNF-α	7	57.5 (44.6 to 69.5) (26.7 to 83.4)	74.5 (67.0 to 80.7) (55.5 to 87.2)	66
WCC	16	60.1 (50.0 to 68.5) (28.5 to 85.0)	68.9 (58.0 to 78.1) (26.0 to 93.3)	64.5
ANC	6	63.4 (54.6 to 71.5) (43.8 to 79.4)	65.4 (48.8 to 78.9) (27.3 to 90.5)	64.4

Pooled estimates and uncertainty intervals were generated to illustrate comparative biomarker performance. PI: 95% PI, indicating the range within which the true effect of a new study is expected to fall. CI: 95% CI, indicating the uncertainty around the pooled mean estimate.

* † defined as (sensitivity+specificity)/2.

ANC, absolute neutrophil count; CRP, C reactive protein; hs-CRP, high sensitivity CRP; IL, interleukin; I/T ratio, immature-to-total leukocyte ratio; MPV, mean platelet volume; NLR, neutrophil-to-lymphocyte ratio; PCT, procalcitonin; PI, prediction interval; PLT, platelet count; SAA, serum amyloid A; sTREM-1, soluble triggering receptor expressed on myeloid cells-1; TNF-α, tumour necrosis factor alpha; WBC, white blood cell.

Many qualitative and quantitative rapid tests (most in lateral flow format) are commercially available for established single and combined biomarkers, including CRP, procalcitonin (PCT), IL-6 and SAA. However, POC diagnostics are more limited for less established, high-performing biomarkers, such as sTREM-1, presepsin and CRP+CD64. More advanced algorithm-based tests for the combined detection of host-response biomarkers that leverage AI are in development but estimated timelines for commercialisation are hard to determine. Achieving improved accuracy will likely require serial or continuous measurements, quantitative outputs, and the validation of multiple biomarker combinations. Advanced diagnostic platforms that integrate host-response signatures with machine learning algorithms represent a significant advance in the field. Platforms such as Inflammatix’s TriVerity system analyse mRNA signatures to predict infection type and severity. TriVerity, which has demonstrated the ability to outperform biomarkers such as WBC, CRP and PCT, generates scores for the likelihood of bacterial or viral infection, as well as the likelihood of requiring intensive interventions such as ventilation, vasopressors or dialysis.^35^ While these technologies hold promise, they have not yet been validated or approved for use in neonates and require further development to ensure improved affordability, usability and suitability for LMIC settings.

While several of the biomarkers show potential, most do not achieve the WHO TPP sensitivity and specificity required for use as standalone diagnostic tools.^34^ The lack of standardised methods and definitions creates considerable heterogeneity among published studies, making it difficult to make comparative conclusions from the data and highlighting the need for a harmonised approach to validate findings across studies. Crucially, there is a lack of data from LMICs, with just 9.5% of neonatal sepsis biomarker research conducted in South Asia and Africa, despite the high burden of neonatal sepsis in these regions.^36^

### Haematological parameters

While biomarkers show promise, their widespread use in LMICs is currently limited by cost, availability and limited adoption in routine care. A more practical approach is to analyse routine haematological parameters using existing laboratory testing or by implementing affordable and environmentally adapted POC tests. Haematological parameters are important for diagnosing neonatal sepsis, as they reflect the activation of the innate immune system, which represents a neonate’s primary early defence against infection. While total WBCs alone are potentially insufficient for diagnosing neonatal sepsis (table 1), analysing a combination of blood cell types and their morphological changes could improve diagnostic accuracy. One widely evaluated prediction models in LMICs is the haematological scoring system.^16^ This model assigns scores to various abnormal parameters, such as WBCs, neutrophil counts (including neutropenia), ratios, abnormal platelet counts, and includes morphological changes such as toxic granulation, Döhle bodies and cytoplasmic vacuolation.^37^ Although these responses are not yet fully developed in neonates, infection triggers the activation, proliferation and depletion of these blood cells. Analysis of several of these parameters has traditionally required the manual analysis of blood smears, which can be labour-intensive and lacks accuracy. Advances in AI-assisted image analysis technology and compact haematology analysers, now available at the POC, including analysers such as PixCell HemoScreen, Dymind DP-H10, HemoCue WBC System and Biosurfit spinit, allow for measurement of WBCs, differentials, platelets, haemoglobin and biomarkers such as CRP and SAA from minimal blood volumes in minutes. These advances could enable measurements at or near the POC, making haematological monitoring a more feasible and cost-effective alternative to standalone biomarker-based diagnostics. Manual peripheral blood smears may still be needed for high-risk neonates, however, emerging AI-assisted smear analysis platforms could improve accuracy and speed up morphological assessment. Similar to host-response biomarkers, the levels of haematological parameters need to be determined and validated at specific times during neonatal development, as these parameters are in constant flux during this period. Integrating these parameters with an AI algorithm could improve their performance, and incorporating these more affordable haematology analysers into routine care can strengthen overall health system capacity, as the same devices could be used for routine laboratory testing across patient populations. However, further research and development are necessary to validate these approaches and ensure their feasibility and implementation in different contexts.

### Continuous monitoring devices

Continuous, non-invasive monitoring of vital signs to detect subtle patterns or gradual physiological changes associated with sepsis ^38^ offers clear advantages over detecting blood-based host-response parameters. Vital signs that can be monitored using existing technologies include temperature, respiratory rate, oxygen saturation (SpO_2_) and heart rate. Infection can disrupt the homeostatic regulation of body temperature, inducing either hypothermia or hyperthermia. While changes in temperature often lack sufficient accuracy alone,^39^ and can be affected by external heat sources, when used alongside other parameters, it can assist in diagnosing neonatal sepsis. There is a body of evidence that sepsis, also via inflammatory mediators, dysregulates a neonate’s respiratory rate, leading to apnoea and associated desaturation as defined by a drop in SpO_2_.^40^,^42^ Changes in heart rate are also an early indicator of sepsis. A study that included automated scoring of physiological signals, including heart rate, respiratory rate and oxygen saturation, reported an area under the curve (AUC) of 0.854 for morbidity, as determined by Receiver Operating Characteristic curves.^43^ While more complex to measure and currently less accessible to LMICs, heart rate characteristics (HRC) and heart rate variability (HRV) may provide more prognostic information than heart rate alone. HRV refers to the variation in time intervals between consecutive heartbeats and is associated with systemic inflammatory responses in sepsis. HRC refers to specific measures of heart rate abnormalities, including reduced beat-to-beat variability and episodes of bradycardia^44^. Advanced digital technology and AI are accelerating the functionality and use of these technologies. Although the literature on the use of continuous monitoring systems in LMICs for neonatal sepsis detection is limited, promising wearable technologies such as those produced by Sibel Health, NeoPenda and Bambi Medical suggest this is becoming increasingly more feasible and affordable. The use of these systems has shown potential to decrease sepsis-associated mortality; however, more validation is still required and challenges such as signal disturbance, compromised circulation and skin damage need to be taken into account.^45^ The use of these data, in combination with other variables that can be measured at the POC and embedded in routine care, may improve overall care in under resourced neonatal wards.

### Early warning scores/trigger and track systems

Many hospitals use standardised scoring systems, for example, the Newborn Early Warning Trigger and Track 2 framework in the UK, that combine multiple vital signs and clinical indicators to identify at-risk neonates, typically through the use of colour-coded scores that can be embedded into paper-based or electronic observation charts. These rely on frequent assessments of vital signs and can occur with or without continuous monitoring which could be challenging in settings with human resource constraints. However, they are not specific for sepsis detection, and evidence for neonatal sepsis trigger tools is limited.^46 47^

### AI in the early detection and prediction of neonatal sepsis

AI is set to transform neonatal sepsis detection, moving beyond single diagnostics and traditional manual scoring systems. Manual scores are not capable of capturing the relationship between various factors, as they assess criteria in a relatively independent manner and thus may overlook combined effects. With access to high-quality data from various technologies, AI-based tools can achieve greater accuracy and predictive performance^47^ . These models can integrate a variety of data, including heart rate, respiratory rate, oxygen saturation, haematological parameters and biomarkers, many of which are now measurable by technologies that are more affordable and available at the POC. This has become even more of a reality with the recent US Food and Drug Administration approval of an AI-based software tool, SepsisImmunoscore, developed by Prenosis Inc.^47^ This tool incorporates parameters from routinely collected clinical data in the USA and uses them to predict the risk that an adult will develop sepsis within the next 24 hours. LMICs are also beginning to leverage AI tools for frontline health workers. Accredited Social Health Activists (ASHAs), who visit neonates up to to 42 days after birth, are using an AI tool to extract key anthropometric measurements from a video in real-time alongside an AI WhatsApp ASHABot that supports and guides the ASHAs with medical information. In Zimbabwe, a group is developing statistical and machine learning algorithms using Neotree, an open-source digital health intervention tool, to triage neonates at risk of sepsis in low-resource settings.^48^ Although the model’s performance does not currently meet the WHO performance thresholds, adding the parameters identified in this review could improve the algorithm’s accuracy to reflect a similar but LMICs-specific algorithm like the SepsisImmunoscore.

## Conclusions

**Figure 2:**
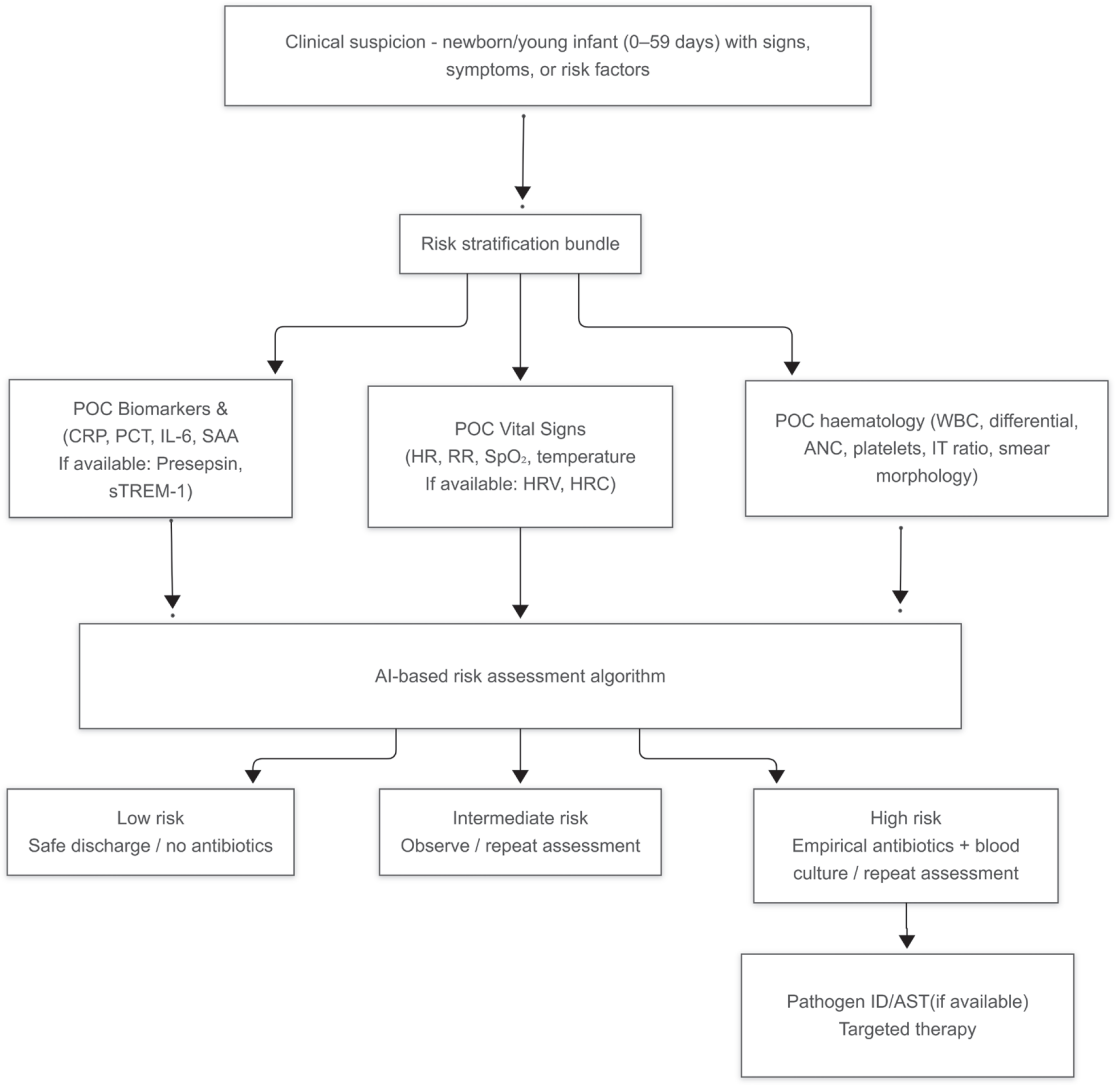
Clinical pathway and risk stratification using POC diagnostics. Proposed PoC clinical pathway for newborns and young infants (0–59 days) with signs, symptoms or risk factors for neonatal sepsis, which includes sampling for host-response biomarkers/haematology parameters and vital signs to be incorporated into an AI-based assessment to classify infants as low, intermediate or high risk and guide treatment decisions. AI, artificial intelligence; ANC, absolute neutrophil count; CRP, C reactive protein; HR, heart rate; HRC, HR characteristics; HRV, HR variability; I/T ratio, immature-to-total neutrophil ratio; PCT, procalcitonin; POC, point of care; RR, respiratory rate; SpO2, peripheral oxygen saturation; sTREM-1, soluble triggering receptor expressed on myeloid cells-1; WBC, white blood cell.

The main challenges for LMICs will not be the need for new technologies, but rather the capacity to integrate existing technologies into fragile health systems. This will include the need for robust connectivity infrastructure and trained healthcare workers. Translating this to the diagnosis of neonatal sepsis in LMICs will require the collection of high-quality data, population-specific validation, and ensuring that the necessary hardware is affordable, accessible, user-friendly and runs on open-source software. Based on the evidence in this review, the ideal triage approach should support clinical judgement by providing risk stratification and could potentially include continuously monitored vital signs that would, on signs of distress or clinical concern, trigger the need for a POC test that measures a biomarker panel that could inculde, for example, SAA and IL-6 and, if available and affordable, presepsin and sTREM-1, as well as haematological parameters including the I/T ratio, absolute neutrophil count and platelet counts (figure 2). The model would then integrate all of these data points to generate an accurate, real-time sepsis risk score that meets the requirements of WHO TPP for high-sensitivity triage, operational feasibility, usability and scalability in LMIC settings. At scale, this bundle of POC technologies could meet the WHO TPP affordability target for LMIC neonatal triage at US$3–US$5 per test (table 2), particularly when instruments are used as part of an integrated neonatal care package rather than as stand-alone sepsis tests.

**Table 2:** Estimated costs for a bundle of POC technologies

POC bundle	Cost per instrument	Cost per test	Examples*	Maintenance (as per examples)
POC lateral flow assay (Quantitative)	Moderate	Low	Boditech iChroma	Minimal/cleaning only. Minimal routine maintenance required.
POC haematology analyser	Moderate	Low	PixCell HemoScreen	Minimal to low. No user maintenance or calibration, cartridge-based.
Combination biomarkers and haematology	Moderate	Low	Biosurfit	Minimal. System is designed to be ‘maintenance-free’, only QC checks required.
Wearables	Low to moderate	Low	Neopenda (Neoguard)	Low. simple, reusable, easy to sanitise, requiring minimal maintenance or servicing.
AI software	No per-test consumables	Low	Prenosis ImmunoScore (LMIC-adapted)	None. Subscription model.

Cost per test: Low <US$5; Moderate US$5–US$20; High >US$5000.

Instrument cost: Low <US$200; Moderate US$200–US$5000; High >US$5000.

* Examples are illustrative and not exhaustive.

AI, artificial intelligence; LMIC, low- and middle-income country; POC, point of care.

## Supplementary material

10.1136/bmjgh-2025-023363 online supplemental file 1

## Data Availability

The data that support the findings of this study are available on request from the corresponding author, BG.
