# Supplementary material for: Aligning neonatal sepsis triage diagnostics with WHO target product profiles in low- and middle-income countries
Source: BMJ Glob Health. 2026 Jun 16;11(6):e023363. doi: 10.1136/bmjgh-2025-023363 (PMC13288953; doi:10.1136/bmjgh-2025-023363)
Supplement: online supplemental file 1 [file bmjgh-11-6-s001.pdf]

A bivariate random-effects meta-regression was performed using R (version 4.2.2; metafor package [Viechtbauer W. Conducting meta-analyses in R with the metafor package. J Statistical Software 2010; 36: 1–48]) to jointly model sensitivity and specificity (and their correlation) for biomarkers evaluated in ≥3 published studies. When multiple estimates were reported per study, the pair yielding the highest balanced accuracy ([sensitivity + specificity]/2) was retained. Pooled sensitivity and specificity were estimated using the rma.mv function with publication specified as a random factor nested within each moderator. Restricted maximum likelihood (REML) estimation was applied to obtain unbiased heterogeneity estimates. Results are presented with 95% confidence intervals (CIs) and 95% prediction intervals (PIs), representing the uncertainty around the pooled mean and the expected range of true outcomes, respectively.

	Study	Country	Design	Type	N (total)	N (test/control)	Biomarker(s)	Control	Confirmation
1	Adib et al., 2012(1)	Iran	Prospective	Case control	87	69 / 18	CRP, PCT	Healthy	Clinical and culture
2	Adly et al., 2014(2)	Egypt	Prospective	Case control	152	112 / 40	hsCRP, sTREM-1	Healthy	Clinical and culture
3	Ahmed et al., 2019(3)	Egypt	Prospective	Case control	60	30 / 30	CRP, IL-8, IL-6, PCT, presepsin	Healthy	Clinical and culture
4	Alima Yanda et al., 2015(4)	Cameroon	Prospective	Cohort	98	25 / 73	PCT	Suspected sepsis, not confirmed	Clinical and culture
5	Alkan Ozdemir et al., 2018(5)	Turkey	Prospective	Cohort	127	52 / 75	CRP, NRL	Suspected sepsis, not confirmed	Culture
6	Alkan Ozdemir et al., 2018(6)	Turkey	Prospective	Cohort	62	31 / 31	sTREM-1	Suspected sepsis, not confirmed	Culture
7	Al-Zahrani et al., 2015(7)	Saudi Arabia	Prospective	Cohort	100	71 / 29	CRP, IL-6, PCT	Suspected sepsis, not confirmed	Clinical and culture
8	Auriti et al., 2012(8)	Italy	Prospective	Cohort	762	65 / 697	PCT	Suspected sepsis, not confirmed	Clinical and culture
9	Ayazi et al., 2014(9)	Iran	Prospective	Cohort	83	16 / 67	CRP	Suspected sepsis, not confirmed	Culture
10	Aydemir et al., 2018(10)	Turkey	Retrospective	Cohort	321	98 / 223	CRP, MVP, PCT	Condition unrelated to sepsis	Culture
11	Badr et al., 2018(11)	Egypt	Prospective	Cohort	38	23 / 15	CRP	Healthy	Culture
12	Basu et al., 2012(12)	India	Prospective	Case control	64	32 / 32	IL-6	Healthy	Clinical and culture

13	Basu et al., 2015(13)	India	Prospective	Case control	64	32 / 32	TNF-α	Healthy	Clinical and culture
14	Beltempo et al., 2018(14)	Canada	Retrospective	Cohort	590	162 / 428	CRP, WBC	Suspected sepsis, not confirmed	Culture
15	Berka et al., 2021(15)	Czech Republic	Retrospective	Cohort	285	66 / 219	CRP, IL-6, PCT	Suspected sepsis, not confirmed	Culture
16	Berka et al., 2022(16)	Czech Republic	Retrospective	Cohort	445	53 / 392	IL-6	Suspected sepsis, not confirmed	Clinical and culture
17	Berrington et al., 2014(17)	United Kingdom	Prospective	Cohort	50	9 / 41	CD64	Suspected sepsis, not confirmed	Culture
18	Bohnhorst et al., 2012(18)	Germany	Prospective	Cohort	170	58 / 112	CRP, IL-6, PCT, CRP + IL-6, CRP + PCT, PCT + IL-6	Suspected infection, not confirmed	Culture
19	Boonkasidecha et al., 2013(19)	Thailand	Prospective	Cohort	53	26 / 27	CRP	Suspected infection, not confirmed	Culture
20	Boskabadi et al., 2013(20)	Iran	Prospective	Case control	84	41 / 43	IL-6	Healthy	Clinical and culture
21	Bourika et al., 2020(21)	Greece	Prospective	Case control	113	45 / 68	CRP, SAA	Healthy	Clinical and culture
22	Bunduki and Adu-Sarkodie, 2020(22)	DRC	Prospective	Cohort	228	69 / 159	CRP	Suspected infection, not confirmed	Culture
23	Cao et al., 2012(23)	China	Prospective	Cohort	356	196 / 160	CRP	Healthy	Clinical and culture
24	Celik et al., 2013(24)	Turkey	Retrospective	Case control	304	206 / 98	IL-6, CRP	Condition unrelated to sepsis	Clinical and culture
25	Chaurasia et al., 2023(25)	India	Prospective	Cohort	855	188 / 667	PCT	Suspected sepsis, not confirmed	Culture
26	Chen et al., 2017(26)	China	Prospective	Cohort	149	96/53	CRP, PCT, Presepsin, WBC	Condition unrelated to sepsis	Clinical and culture
27	Choo et al., 2012(27)	Korea	Prospective	Cohort	23	11 / 12	CRP, CD64, WBC, ANC	Suspected sepsis, not confirmed	Culture
28	Coly et al., 2021(28)	Senegal	Prospective	Cohort	99	26 / 73	CRP, IL-6, PCT, SAA, IL-8	Suspected sepsis, not confirmed	Clinical and culture

29	Cortés et al., 2021(29)	Colombia	Prospective	Case control	93	31 / 62	CRP, IL-6	Suspected sepsis, not confirmed	Clinical
30	Dhudasia et al., 2023(30)	USA	Retrospective	Cohort	6193	30 / 6163	CRP	Suspected sepsis, not confirmed	Culture
31	Dierikx et al., 2023(31)	Netherlands	Prospective	Cohort	169	15 / 154	Presepsin	Suspected sepsis, not confirmed	Clinical and culture
32	Dillenseger et al., 2018(32)	France	Prospective	Cohort	130	34 / 96	CRP, IL-8, PCT, CRP + IL-6	Suspected sepsis, not confirmed	Clinical and culture
33	Du et al., 2014[42](33)	China	Prospective	Cohort	158	88 / 70	CD64, WBC, CRP, ANC, CRP + CD 64	Healthy	Clinical
34	Du et al., 2016(34)	China	Prospective	Cohort	157	79 / 78	CRP, PCT, WBC	Suspected sepsis, not confirmed	Clinical and culture
35	Ebenebe et al., 2019(35)	Germany	Retrospective	Case control	182	67/115	Il-6	Condition unrelated to sepsis	Clinical and culture
36	El Shimi et al., 2017(36)	Egypt	Prospective	Case control	90	60 / 30	hsCRP, CD64	Healthy	Clinical and culture
37	Elawady et al., 2014(37)	Egypt	Prospective	Cohort	75	50 / 25	CD64, WBC, PLT, I/T ratio, ANC	Condition unrelated to sepsis	Clinical and culture
38	El-Madbouly et al., 2019(38)	Egypt	Prospective	Case control	60	30 / 30	Presepsin, sTREM-1, CD64, CRP, CRP + CD64	Healthy	Clinical and culture
39	Fahmey and Mostafa, 2019(39)	Egypt	Prospective	Case control	90	60 / 30	CRP	Healthy	Culture
40	Fattah et al., 2017(40)	Saudi Arabia	Prospective	Cohort	320	240 / 80	CRP, TNFα, PCT, CRP + IL-6, CRP + PCT, PCT + IL-6	Healthy	Clinical and culture
41	Ganesan et al., 2016(41)	India	Prospective	Case control	80	40 / 40	CRP, IL-6, hsCRP	Healthy	Clinical and culture
42	Gao et al., 2021(42)	China	Prospective	Cohort	142	63 / 79	hsCRP, PLT	Condition unrelated to sepsis	Culture
43	Gatseva et al., 2023(43)	Bulgaria	Prospective	Case control	56	21 / 35	PCT, IL-6, PLT, I/T ratio, PCT + IL-6	Condition unrelated to sepsis / Suspected sepsis, not confirmed	Clinical and culture

44	Go et al., 2020(44)	Japan	Retrospective	Cohort	23	6 / 17	PCT, CRP, WBC	Suspected sepsis, not confirmed	Clinical and culture
45	Goswami et al., 2022(45)	India	Prospective	Cohort	100	34 / 66	CD64	Suspected sepsis, not confirmed	Culture
46	Habib et al., 2021(46)	Pakistan	Prospective	Cohort	171	86 / 85	PCT	Suspected sepsis, not confirmed	Culture
47	Hahn et al., 2018(47)	South Korea	Prospective	Case control	318	106 / 212	CRP, PCT	Healthy	Clinical and culture
48	Hashem et al., 2020(48)	Egypt	Prospective	Case control	121	91 / 30	CD64, hsCRP, ANC, I/T ratio	Condition unrelated to sepsis	Clinical and culture
49	Hashem et al., 2020(49)	Egypt	Prospective	Case control	235	133 / 102	CD64, presepsin, ANC, hsCRP, CRP + CD64	Healthy/Condition unrelated to sepsis	Clinical and culture
50	He et al., 2017(50)	China	Prospective	Cohort	151	68 / 83	IL-6, IL-8, TNFα, PCT, CRP	Suspected sepsis, not confirmed	Clinical and culture
51	Hibbert et al., 2021(51)	Australia	Retrospective	Cohort	49	28 / 21	IL-6	Suspected sepsis, not confirmed	Clinical and culture
52	Karabulut and Arcagok, 2020(52)	Turkey	Retrospective	Cohort	159	67 / 92	CRP, PCT, I/T ratio	Healthy	Culture
53	Kaur and Singh, 2021(53)	India	Prospective	Cohort	60	16 / 44	CRP	Suspected sepsis, not confirmed	Culture
54	Khan, 2019(54)	Pakistan	Prospective	Cohort	385	335 / 50	CRP	Suspected sepsis, not confirmed	Culture
55	Khattab et al., 2018 (55)	Egypt	Prospective	Cohort	90	60 / 30	CRP, WBC, ANC, I/T ratio, NLR, PLT	Healthy	Clinical and culture
56	Kim et al., 2015(56)	South Korea	Retrospective	Cohort	292	32 / 260	MVP, PLT	Healthy	Clinical and culture
57	Kordek et al., 2014(57)	Poland	Prospective	Cohort	140	52 / 88	CRP, PCT, WBC	Condition unrelated to sepsis	Clinical and culture
58	Kumar et al., 2018(58)	India	Prospective	Case control	82	41 / 41	CRP, PCT, presepsin	Condition unrelated to sepsis	Clinical and culture
59	Kung et al., 2023(59)	Austria	Retrospective	Cohort	1036	927 / 109	IL-6	Healthy	Culture
60	Li et al., 2020(60)	China	Retrospective	Case control	925	737 / 188	NLR	Condition unrelated to sepsis	Clinical

61	Li et al., 2013(61)	China	Prospective	Cohort	111	26 / 85	CRP	Suspected sepsis, not confirmed	Clinical and culture
62	Liu et al., 2020(62)	China	Retrospective	Cohort	110	56 / 54	CRP, PCT	Suspected sepsis, not confirmed	Clinical and culture
63	Liu et al., 2020(63)	China	Prospective	Cohort	152	102 / 50	PCT	Condition unrelated to sepsis	Clinical and culture
64	Liu et al., 2020(64)	China	Retrospective	Cohort	129	66 / 63	CRP, IL-6, PCT, SAA	Healthy	Clinical and culture
65	Liu et al., 2020(65)	China	Retrospective	Case control	472	169/303	Albumin	Healthy	Culture
66	Lusyati et al., 2013(66)	Indonesia	Prospective	Cohort	52	18 / 34	IL 6, IL-8, TNFα	Condition unrelated to sepsis	Culture
67	Mazzucchelli et al., 2013(67)	Italy	Prospective	Case control	32	16 / 16	CD64	Healthy/Condition unrelated to sepsis	Culture
68	Miyosawa et al., 2018(68)	Japan	Prospective	Case control	31	13 / 18	Presepsin	Healthy	Culture
69	Mkony et al., 2014(69)	Tanzania	Prospective	Cohort	208	40 / 168	CRP	Suspected sepsis, not confirmed	Culture
70	Mohsen and Kamel, 2015(70)	Egypt	Prospective	Case control	70	35 / 35	CRP, PCT	Healthy	Clinical and culture
71	Monsef and Eghbalian, 2012(71)	Iran	Prospective	Cohort	71	39 / 32	PCT	Healthy/Condition unrelated to sepsis	Clinical and culture
72	Montaldo et al., 2017(72)	Italy	Prospective	Case control	70	32 / 38	CRP, PCT, Presepsin	Healthy/Condition unrelated to sepsis	Culture
73	Morad et al., 2020(73)	Egypt	Prospective	Cohort	50	41 / 9	CRP, IL-6, PCT	Suspected sepsis, not confirmed	Clinical and culture
74	Motalib et al., 2015(74)	Egypt	Prospective	Case control	62	28/34	CRP, Presepsin	Healthy	Clinical and culture
75	Motta et al., 2014(75)	Italy	Prospective	Cohort	129	48 / 81	CD64	Suspected sepsis, not confirmed	Clinical and culture
76	Mussap et al., 2015(76)	Italy	Prospective	Cohort	50	25 / 25	Presepsin	Condition unrelated to sepsis	Culture
77	Naramura et al., 2021(77)	Japan	Retrospective	Cohort	320	39/281	hsCRP, PCT	Healthy/Condition unrelated to sepsis	Clinical and culture

78	Omar et al., 2019(78)	Malaysia	Prospective	Cohort	60	12 / 48	PCT	Suspected sepsis, not confirmed	Culture
79	Omran et al., 2021(79)	Egypt	Prospective	Case control	70	35 / 35	IL-10, MPV	Condition unrelated to sepsis	Clinical and culture
80	Oncel et al., 2012(80)	Turkey	Prospective	Case control	83	31 / 52	CRP, IL-6, WBC	Healthy	Culture
81	Osman et al., 2015 (81)	Egypt	Prospective	Case control	55	40/15	Presepsin	Healthy	Clinical and culture
82	Ozdemir and Elgormus, 2017(82)	Turkey	Prospective	Cohort	69	29 / 40	CRP, PCT, presepsin	Condition unrelated to sepsis	Clinical and culture
83	Panda et al., 2021(83)	India	Retrospective	Case control	93	41 / 52	NLR, CRP	Condition unrelated to sepsis	Culture
84	Park et al., 2014(84)	Korea	Retrospective	Cohort	269	155 / 114	PCT, CRP	Suspected sepsis, not confirmed	Clinical and culture
85	Poggi et al., 2015(85)	Italy	Prospective	Cohort	40	19 / 21	Presepsin	Condition unrelated to sepsis; Suspected sepsis, not confirmed	Clinical and culture
86	Pospisilova et al., 2023(86)	Czech Republic	Prospective	Cohort	101	20 / 81	Presepsin	Suspected sepsis, not confirmed	Clinical
87	Prashant et al., 2013(87)	India	Prospective	Cohort	150	50 / 100	CRP, IL-6, IL-8, TNFα	Suspected sepsis, not confirmed	Culture
88	Pravin Charles et al., 2018(88)	India	Prospective	Cohort	75	56 / 19	CRP, PCT	Suspected sepsis, not confirmed	Clinical and culture
89	Puello Ávila et al., 2021(89)	Colombia	Prospective	Cohort	198	33 / 165	CRP	Suspected sepsis, not confirmed	Clinical and culture
90	Rao et al., 2020(90)	China	Prospective	Cohort	117	53 / 64	PCT, CRP, CRP + PCT	Suspected sepsis, not confirmed	Clinical and culture
91	Rashwan et al., 2019(91)	Egypt	Prospective	Cohort	168	102 / 66	hsCRP, CRP, presepsin, PCT	Suspected sepsis, not confirmed	Culture
92	Rass et al., 2016(92)	Egypt	Prospective	Cohort	104	52 / 52	CRP	Suspected sepsis, not confirmed	Clinical and culture
93	Raynor et al., 2012(93)	USA	Retrospective	Cohort	226	128 / 98	CRP, IL-6	Suspected sepsis, not confirmed	Clinical and culture

94	Rohsiswatmo et al., 2021(94)	Indonesia	Prospective	Cohort	52	17 / 35	I/T ratio, CRP, PCT, WBC	Suspected sepsis, not confirmed	Clinical and culture
95	Rosenfeld et al., 2019(95)	USA	Retrospective	Cohort	100	54 / 46	I/T ratio	Suspected sepsis, not confirmed	Culture
96	Saldir et al., 2015(96)	Turkey	Prospective	Cohort	50	30 / 20	IL-6, sTREM-1, I/T ratio	Suspected sepsis, not confirmed	Clinical and culture
97	Schlapbach et al., 2013(97)	Switzerland	Prospective	Cohort	137	33 / 104	PCT, sTREM-1, CRP, I/T ratio, WBC	Suspected sepsis, not confirmed	Clinical and culture
98	Sethi et al., 2023 (98)	India	Prospective	Cohort	100	33 / 67	PCT, hsCRP, CRP	Suspected sepsis, not confirmed	Culture
99	Shi et al., 2023(99)	China	Retrospective	Cohort	2049	732 / 1317	CRP, PCT, WBC, Albumin, CRP + PCT	Suspected sepsis, not confirmed	Clinical and culture
100	Sorsa 2018(100)	Ethiopia	Prospective	Cohort	303	88 / 215	WBC, CRP	Suspected sepsis, not confirmed	Culture
101	Streimish et al., 2012(101)	USA	Prospective	Cohort	583	3/580	CD64	Suspected sepsis, not confirmed	Culture
102	Sumitro et al., 2021(102)	Indonesia	Prospective	Cohort	104	52 / 52	NLR	Suspected sepsis, not confirmed	Culture
103	Tang et al., 2022 (103)	Taiwan	Retrospective	Cohort	123	4 / 119	PCT, CRP, PLT, CRP + PCT	Suspected sepsis, not confirmed	Culture
103	Tang et al., 2022(103)	Taiwan	Retrospective	Cohort	46	8 / 38	WBC	Suspected sepsis, not confirmed	Culture
104	Topcuoglu et al., 2016(104)	Turkey	Prospective	Case control	82	42 / 40	Presepsin	Condition unrelated to sepsis	Clinical and culture
105	Tosson et al., 2020(105)	Egypt	Prospective	Cohort	118	74 / 44	IL-6, IL-10	Condition unrelated to sepsis	Clinical and culture
106	Tunc et al., 2015(106)	Turkey	Prospective	Cohort	50	30 / 20	IL-6, I/T ratio	Condition unrelated to sepsis	Clinical and culture
107	Wilar, 2019(107)	Indonesia	Prospective	Cohort	120	90 / 30	NLR	Suspected sepsis, not confirmed	Clinical and culture
108	Wu et al., 2013(108)	USA	Prospective	Cohort	44	17 / 27	CRP	Suspected sepsis, not confirmed	Clinical and Culture

119	Xiao et al., 2017 (a)(109)	China	Retrospective	Cohort	298	192 / 106	Presepsin, CRP	Healthy	Clinical and culture
110	Xiao et al., 2017 (b)(110)	China	Prospective	Cohort	193	42 / 151	Presepsin, PCT, CRP, WBC	Healthy	Culture
111	Yang et al., 2020(111)	China	Prospective	Cohort	152	76 / 76	IL-6, TNFα, PCT, CRP	Suspected sepsis, not confirmed	Clinical and culture
112	Ye et al., 2017(112)	China	Prospective	Case control	840	420 / 420	IL-6, IL-10, TNFα, CRP	Healthy/Condition unrelated to sepsis	Culture
113	Yin et al., 2024(113)	China	Retrospective	Case control	160	80 / 80	CRP, Albumin	Healthy	Clinical and culture

1. Adib M, Bakhshiani Z, Navaei F, Fosoul FS, Fouladi S. Procalcitonin: A Reliable Marker for the Diagnosis of Neonatal Sepsis.

2. Adly AAM, Ismail EA, Andrawes NG, El-Saadany MA. Circulating soluble triggering receptor expressed on myeloid cells-1 (sTREM-1) as diagnostic and prognostic marker in neonatal sepsis. Cytokine. 2014 Feb;65(2):184–91.

3. Ahmed AM, Mohammed AT, Bastawy S, Attalla HA, Yousef AA, Abdelrazek MS, et al. Serum Biomarkers for the Early Detection of the Early-Onset Neonatal Sepsis: A Single-Center Prospective Study. Advances in Neonatal Care. 2019 Oct;19(5):E26–32.

4. Alima Yanda AN, Kobela M, Nansseu JR, Taguebue J, Boula A, Eposse C, et al. Intérêt du dosage de la procalcitonine sérique dans le diagnostic et le suivi des infections néonatales d'origine bactérienne à Yaoundé, Cameroun. Archives de Pédiatrie. 2015 Oct;22(10):1015–20.

5. Alkan Ozdemir S, Arun Ozer E, Ilhan O, Sutcuoglu S. Can neutrophil to lymphocyte ratio predict late-onset sepsis in preterm infants? Clinical Laboratory Analysis. 2018 May;32(4):e22338.

6. Alkan Ozdemir S, Ozer EA, Ilhan O, Sutcuoglu S, Tatlı M. Diagnostic value of urine soluble triggering receptor expressed on myeloid cells (sTREM-1) for late-onset neonatal sepsis in infected preterm neonates. J Int Med Res. 2018 Apr;46(4):1606–16.

7. Al-Zahrani AK, Ghonaim MM, Hussein YM, Eed EM, Khalifa AS, Dorgham LS. Evaluation of recent methods versus conventional methods for diagnosis of early-onset neonatal sepsis. J Infect Dev Ctries. 2015 Mar 15;9(04):388–93.

8. Auriti C, Fiscarelli E, Ronchetti MP, Argentieri M, Marrocco G, Quondamcarlo A, et al. Procalcitonin in detecting neonatal nosocomial sepsis.

9. Ayazi P, Mahyar A, Daneshi MM, Jahanihashemi H, Esmailzadehha N, Mosaferirad N. Comparison of serum IL-1beta and C-reactive protein levels in early diagnosis and management of neonatal sepsis.

10. Aydemir C, Aydemir H, Kokturk F, Kulah C, Mungan AG. The cut-off levels of procalcitonin and C-reactive protein and the kinetics of mean platelet volume in preterm neonates with sepsis. BMC Pediatr. 2018 Dec;18(1):253.

11. Badr HS, El-Gendy FM, Helwa MA. Serum stromal-derived-factor-1 (CXCL12) and its alpha chemokine receptor (CXCR4) as biomarkers in neonatal sepsis. The Journal of Maternal-Fetal & Neonatal Medicine. 2018 Aug 18;31(16):2209–15.

12. Basu S. Elevated plasma and cerebrospinal fluid interleukin-1 beta and tumor necrosis factor-alpha concentration and combined outcome of death or abnormal neuroimaging in preterm neonates with early-onset clinical sepsis. Journal of Perinatology. 2015;

13. Basu S, Dewangan S, Shukla RC, Anupurva S, Kumar A. Thymic involution as a predictor of early-onset neonatal sepsis.

14. Beltempo M. C-reactive protein for late-onset sepsis diagnosis in very low birth weight infants.

15. Berka I, Korček P, Straňák Z. C-Reactive Protein, Interleukin-6, and Procalcitonin in Diagnosis of Late-Onset Bloodstream Infection in Very Preterm Infants. Journal of the Pediatric Infectious Diseases Society. 2021 Aug 3;piab071.

16. Berka I, Korček P, Straňák Z. Serial Measurement of Interleukin-6 Enhances Chance to Exclude Early-Onset Sepsis in Very Preterm Infants. Clin Pediatr (Phila). 2023 May;62(4):288–94.

17. Berrington JE, Hearn RI, Hall C, Stewart CJ, Cummings SP, Embleton ND. Proportionate Reduction in Uncertainty of Late Onset Infection in Pre-term Infants by Neutrophil CD64 Measurement. Fetal and Pediatric Pathology. 2014 Feb;33(1):16–22.

18. Bohnhorst B, Lange M, Bartels DB, Bejo L, Hoy L, Peter C. Procalcitonin and valuable clinical symptoms in the early detection of neonatal late-onset bacterial infection. Acta Paediatrica. 2012 Jan;101(1):19–25.

19. Boonkasidecha S, Panburana J, Chansakulporn S, Benjasuwantep B, Kongsomboon K. An optimal cut-off point of serum C-reactive protein in prediction of neonatal sepsis. J Med Assoc Thai. 2013 Jan;96 Suppl 1:S65-70.

20. Boskabadi H, Maamouri G, Afshari JT, Mafinejad S, Mostafavi-Toroghi H, Saber H, et al. Evaluation of Serum Interleukins-6, 8 and 10 Levels as Diagnostic Markers of Neonatal Infection and Possibility of Mortality. Iran J Basic Med Sci. 2013;16(12).

21. Bourika V, Hantzi E, Michos A, Margeli A, Papassotiriou I, Siahanidou T. Clinical Value of Serum Amyloid-A Protein, High-density Lipoprotein Cholesterol and Apolipoprotein-A1 in the Diagnosis and Follow-up of Neonatal Sepsis. Pediatric Infectious Disease Journal. 2020 Aug;39(8):749–55.

22. Bunduki GK, Adu-Sarkodie Y. The usefulness of C-reactive protein as a biomarker in predicting neonatal sepsis in a sub-Saharan African region. BMC Res Notes. 2020 Dec;13(1):194.

23. Cao Y, Xia Q, Chen C, Yang Y. Precursors of adrenomedullin, endothelin and atrial natriuretic peptide as diagnostic markers of neonatal infection. Acta Paediatrica. 2012 Mar;101(3):242–6.

24. Celik IH, Demirel G, Sukhachev D, Erdeve O, Dilmen U. Neutrophil volume, conductivity and scatter parameters with effective modeling of molecular activity statistical program gives better results in neonatal sepsis. Int J Lab Hematology. 2013 Feb;35(1):82–7.

25. Chaurasia S, Anand P, Sharma A, Nangia S, Sivam A, Jain K, et al. Procalcitonin for Detecting Culture-Positive Sepsis in Neonates: A Prospective, Multicenter Study. Neonatology. 2023;120(5):642–51.

26. Chen L, Xiao T, Luo Y, Qiu Q, Que R, Huang X, et al. Soluble CD14 subtype (sCD14-ST) is a biomarker for neonatal sepsis.

27. Choo YK, Cho HS, Seo IB, Lee HS. Comparison of the accuracy of neutrophil CD64 and C-reactive protein as a single test for the early detection of neonatal sepsis. Korean J Pediatr. 2012;55(1):11.

28. Coly NFG, Durif J, Bass I, Pereira B, Thiam S, Samba A, et al. Blood biomarkers of early diagnosis for neonatal bacterial infections: back from Senegal cohort. Annales de Biologie Clinique. 2021 June;79(3):241–52.

29. Cortés JS, Losada PX, Fernández LX, Beltrán E, DeLaura I, Narváez CF, et al. Interleukin-6 as a Biomarker of Early-Onset Neonatal Sepsis. Am J Perinatol. 2021 Aug;38(S 01):e338–46.

30. Dhudasia MB, Benitz WE, Flannery DD, Christ L, Rub D, Remaschi G, et al. Diagnostic Performance and Patient Outcomes With C-Reactive Protein Use in Early-Onset Sepsis Evaluations. The Journal of Pediatrics. 2023 May;256:98-104.e6.

31. Dierikx TH, Van Laerhoven H, Van Der Schoor SRD, Nusman CM, Lutterman CAM, Vliegenthart RJS, et al. Can Presepsin Be Valuable in Reducing Unnecessary Antibiotic Exposure after Birth? Antibiotics. 2023 Apr 2;12(4):695.

32. Dillenseger L, Langlet C, Iacobelli S, Lavaux T, Ratomponirina C, Labenne M, et al. Early Inflammatory Markers for the Diagnosis of Late-Onset Sepsis in Neonates: The Nosodiag Study. Front Pediatr. 2018 Nov 13;6:346.

33. Du J, Li L, Dou Y, Li P, Chen R, Liu H. Diagnostic Utility of Neutrophil CD64 as a Marker for Early-Onset Sepsis in Preterm Neonates. Levy O, editor. PLoS ONE. 2014 July 17;9(7):e102647.

34. Du WX, He Y, Jiang HY, Ai Q, Yu JL. Interleukin 35: A novel candidate biomarker to diagnose early onset sepsis in neonates. Clinica Chimica Acta. 2016 Nov;462:90–5.

35. Ebenebe CU, Hesse F, Blohm ME, Jung R, Kunzmann S, Singer D. Diagnostic accuracy of interleukin-6 for early-onset sepsis in preterm neonates. The Journal of Maternal-Fetal & Neonatal Medicine. 2021 Jan 17;34(2):253–8.

36. El Shimi MS, Abou Shady NM, Hamed GM, Shedeed NS. Significance of neutrophilic CD64 as an early marker for detection of neonatal sepsis and prediction of disease outcome. The Journal of Maternal-Fetal & Neonatal Medicine. 2017 July 18;30(14):1709–14.

37. Elawady S, Botros SK, Sorour AE, Ghany EA, Elbatran G, Ali R. Neutrophil CD64 as a Diagnostic Marker of Sepsis in Neonates. Journal of Investigative Medicine. 2014 Mar;62(3):644–9.

38. El-Madbouly A, El Sehemawy A, Eldesoky N, Abd Elgalil HM, Ahmed A. Utility of presepsin, soluble triggering receptor expressed on myeloid cells-1, and neutrophil CD64 for early detection of neonatal sepsis. IDR. 2019 Jan;Volume 12:311–9.

39. Fahmey SS, Mostafa N. Pentraxin 3 as a novel diagnostic marker in neonatal sepsis. Journal of Neonatal-Perinatal Medicine. 2020 Jan 4;12(4):437–42.

40. Fattah M, Omer AF, Asaif S, Manlulu R, Karar T, Ahmed A, et al. Utility of cytokine, adhesion molecule and acute phase proteins in early diagnosis of neonatal sepsis. J Nat Sc Biol Med. 2017;8(1):32.

41. Ganesan P. Evaluation of IL-6, CRP and hs-CRP as Early Markers of Neonatal Sepsis. JCDR [Internet]. 2016 [cited 2025 Oct 21]; Available from: http://jcdr.net/article_fulltext.asp?issn=0973-709x&year=2016&volume=10&issue=5&page=DC13&issn=0973-709x&id=7764

42. Gao C, Feng Z, Wang L, Zhao X, Fu K, Ma S, et al. The potential value of plasma receptor interacting protein 3 in neonates with culture-positive late-onset sepsis. BMC Infect Dis. 2021 Dec;21(1):919.

43. Gatseva P, Blazhev A, Yordanov Z, Atanasova V. Early Diagnostic Markers of Late-Onset Neonatal Sepsis. Pediatric Reports. 2023 Sept 20;15(3):548–59.

44. Go H, Nagano N, Katayama D, Akimoto T, Imaizumi T, Aoki R, et al. Diagnostic Accuracy of Biomarkers for Early-Onset Neonatal Bacterial Infections: Evaluation of Serum Procalcitonin Reference Curves. Diagnostics. 2020 Oct 18;10(10):839.

45. Goswami S, Gupta R, Ramji S. Flow Cytometry: An Important Diagnostic Tool in Critically Ill Preterm Neonates with Suspected Sepsis. Am J Perinatol. 2022 Apr;39(06):616–22.

46. Habib A, Raza S, Ali U, Zubairi AM, Salim E. Diagnostic Accuracy of Serum Procalcitonin (PCT) as an Early Biomarker of Neonatal Sepsis using Blood Culture as Gold Standard. J Coll Physicians Surg Pak. 2021 Apr;31(4):383–7.

47. Hahn WH, Song JH, Kim H, Park S. Is procalcitonin to C-reactive protein ratio useful for the detection of late onset neonatal sepsis? The Journal of Maternal-Fetal & Neonatal Medicine. 2018 Mar 19;31(6):822–6.

48. Hashem HE, El Masry SA, Mokhtar AM, Ismail EA, Abdelaal NM. Valuable Role of Neutrophil CD64 and Highly Sensitive CRP Biomarkers for Diagnostic, Monitoring, and Prognostic Evaluations of Sepsis Patients in Neonatal ICUs. Serra R, editor. BioMed Research International. 2020 Jan;2020(1):6214363.

49. Hashem HE, Abdel Halim RM, El Masry SA, Mokhtar AM, Abdelaal NM. The Utility of Neutrophil CD64 and Presepsin as Diagnostic, Prognostic, and Monitoring Biomarkers in Neonatal Sepsis. Li Z, editor. International Journal of Microbiology. 2020 Nov 1;2020:1–13.

50. He Y, Du WX, Jiang HY, Ai Q, Feng J, Liu Z, et al. Multiplex Cytokine Profiling Identifies Interleukin-27 as a Novel Biomarker For Neonatal Early Onset Sepsis. Shock. 2017 Feb;47(2):140–7.

51. Hibbert J, Armstrong NJ, Granland C, Ng S, Simmer K, Richmond P, et al. Plasma secretory phospholipase A2 as an early marker for late-onset sepsis in preterm infants—a pilot study. Acta Paediatrica. 2021 Nov;110(11):3011–3.

52. Karabulut B, Arcagok BC. New Diagnostic Possibilities for Early Onset Neonatal Sepsis: Red Cell Distribution Width to Platelet Ratio. Fetal and Pediatric Pathology. 2020 July 3;39(4):297–306.

53. Kaur S, Singh K. Early-Onset Neonatal Sepsis: Role of C-Reactive Protein, Micro-ESR, and Gastric Aspirate for Polymorphs as Screening Markers. Robert AA, editor. International Journal of Pediatrics. 2021 Dec 2;2021:1–6.

54. Khan F. C-reactive Protein as a Screening Biomarker in Neonatal Sepsis. J Coll Physicians Surg Pak. 2019 Oct 1;29(10):951–3.

55. Khattab AA, El-Mekkawy MS, Helwa MA, Omar ES. Utility of serum resistin in the diagnosis of neonatal sepsis and prediction of disease severity in term and late preterm infants. Journal of Perinatal Medicine. 2018 Oct 25;46(8):919–25.

56. Kim JY, Yoon J, Lim CS, Choi BM, Yoon SY. Clinical significance of platelet-associated hematological parameters as an early supplementary diagnostic tool for sepsis in thrombocytopenic very-low-birth-weight infants. Platelets. 2015 Oct 3;26(7):620–6.

57. Kordek A, Łoniewska B, Podraza W, Nikodemski T, Rudnicki J. Usefulness of estimation of blood procalcitonin concentration versus C-reactive protein concentration and white blood cell count for therapeutic monitoring of sepsis in neonates. Postepy Hig Med Dosw. 2014 Dec 21;68:1516–23.

58. Kumar N, Dayal R, Singh P, Pathak S, Pooniya V, Goyal A, et al. A Comparative Evaluation of Presepsin with Procalcitonin and CRP in Diagnosing Neonatal Sepsis. Indian J Pediatr. 2019 Feb;86(2):177–9.

59. Küng E, Unterasinger L, Waldhör T, Berger A, Wisgrill L. Cut-off values of serum interleukin-6 for culture-confirmed sepsis in neonates. Pediatr Res. 2023 June;93(7):1969–74.

60. Li T, Dong G, Zhang M, Xu Z, Hu Y, Xie B, et al. Association of Neutrophil–Lymphocyte Ratio and the Presence of Neonatal Sepsis. Milito C, editor. Journal of Immunology Research. 2020 Dec 2;2020:1–8.

61. Li Y, Li X, Zhou X, Yan J, Zhu X, Pan J, et al. Impact of sepsis on the urinary level of interleukin-18 and cystatin C in critically ill neonates. Pediatr Nephrol. 2013 Jan;28(1):135–44.

62. Liu C, Fang C, Xie L. Diagnostic utility of procalcitonin as a biomarker for late-onset neonatal sepsis. Transl Pediatr. 2020 June;9(3):237–42.

63. Liu G, Liu W, Guo J. Clinical significance of miR-181a in patients with neonatal sepsis and its regulatory role in the lipopolysaccharide-induced inflammatory response. Exp Ther Med [Internet]. 2020 Jan 2 [cited 2025 Oct 21]; Available from: http://www.spandidos-publications.com/10.3892/etm.2020.8408

64. Liu C, Fang C, He Q, Xie L. The value of interleukin-6 (IL-6) within 6 hours after birth in the prompt diagnosis of early-onset neonatal sepsis. Transl Pediatr. 2020 Oct;9(5):629–35.

65. Liu Y, Chai Y, Rong Z, Chen Y. Prognostic Value of Ionized Calcium Levels in Neonatal Sepsis. Ann Nutr Metab. 2020;76(3):193–200.

66. Lusyati S, Hulzebos CV, Zandvoort J, Sukandar H, Sauer PJJ. Cytokines patterns in newborn infants with late onset sepsis. Journal of Neonatal-Perinatal Medicine. 2013;6(2):153–63.

67. Mazzucchelli I, Garofoli F, Ciardelli L, Borghesi A, Tzialla C, Di Comite A, et al. Diagnostic Performance of Triggering Receptor Expressed on Myeloid Cells-1 and CD64 Index as Markers of Sepsis in Preterm Newborns: Pediatric Critical Care Medicine. 2013 Feb;14(2):178–82.

68. Miyosawa Y, Akazawa Y, Kamiya M, Nakamura C, Takeuchi Y, Kusakari M, et al. Presepsin as a predictor of positive blood culture in suspected neonatal sepsis. Pediatrics International. 2018 Feb;60(2):157–61.

69. Mkony MF, Mizinduko MM, Massawe A, Matee M. Management of neonatal sepsis at Muhimbili National Hospital in Dar es Salaam: diagnostic accuracy of C – reactive protein and newborn scale of sepsis and antimicrobial resistance pattern of etiological bacteria. BMC Pediatr. 2014 Dec;14(1):293.

70. Mohsen AHA, Kamel BA. Predictive values for procalcitonin in the diagnosis of neonatal sepsis. Electron Physician. 2015 Aug;7(4):1190–5.

71. Monsef A, Eghbalian F. Evaluation of Diagnostic Value of Procalcitonin as a Marker of Neonatal Bacterial Infections. Iran J Pediatr. 2012;22(3).

72. Montaldo P, Rosso R, Santantonio A, Chello G, Giliberti P. Presepsin for the detection of early-onset sepsis in preterm newborns. Pediatr Res. 2017 Feb;81(2):329–34.

73. Morad EA, Rabie RA, Almalky MA, Gebriel MG. Evaluation of Procalcitonin, C-Reactive Protein, and Interleukin-6 as Early Markers for Diagnosis of Neonatal Sepsis. International Journal of Microbiology. 2020 Oct 1;2020:1–9.

74. Motalib T, Khalaf F, Hendawy GRE, Kotb SE, Ali A, Sharnoby AE. Soluble CD 14-subtype (Prespsin) and Hepcidin as Diagnostic and Prognostic markers in Early Onset Neonatal Sepsis. In 2015 [cited 2025 Oct 21]. Available from: https://www.semanticscholar.org/paper/Soluble-CD-14-subtype-(-Prespsin-)-and-Hepcidin-as-Motalib-Khalaf/aa79bf82a74944bc65653e63a6ccbc92ba8e6b9e

75. Motta M, Zini A, Regazzoli A, Garzoli E, Chirico G, Caimi L, et al. Diagnostic accuracy and prognostic value of the CD64 index in very low birth weight neonates as a marker of early-onset sepsis. Scandinavian Journal of Infectious Diseases. 2014 June;46(6):433–9.

76. Mussap M, Puxeddu E, Puddu M, Ottonello G, Coghe F, Comite P, et al. Soluble CD14 subtype (sCD14-ST) presepsin in premature and full term critically ill newborns with sepsis and SIRS. Clinica Chimica Acta. 2015 Dec;451:65–70.

77. Naramura T, Imamura H, Yoshimatsu H, Hirashima K, Irie S, Inoue T, et al. The Predictive Value of Procalcitonin and High-Sensitivity C-Reactive Protein for Early Bacterial Infections in Preterm Neonates. Neonatology. 2021;118(1):28–36.

78. Omar J, Isa S, Ismail TST, Yaacob NM, Soh NAAC. Procalcitonin as an Early Laboratory Marker of Sepsis in Neonates: Variation in Diagnostic Performance and Discrimination Value. Malays J Med Sci. 2019 July;26(4):61–9.

79. Omran A, Sobh H, Abdalla MO, El-Sharkawy S, Rezk AR, Khashana A. Salivary and Serum Interleukin-10, C-Reactive Protein, Mean Platelet Volume, and CRP/MPV Ratio in the Diagnosis of Late-Onset Neonatal Sepsis in Full-Term Neonates. Duan L, editor. Journal of Immunology Research. 2021 Oct 12;2021:1–7.

80. Oncel MY, Dilmen U, Erdeve O, Ozdemir R, Calisici E, Yurttutan S, et al. Proadrenomedullin as a prognostic marker in neonatal sepsis. Pediatr Res. 2012 Nov;72(5):507–12.

81. Osman AS, Awadallah MG, Tabl HAEM. Presepsin as a Novel Diagnostic Marker in Neonatal Septicemia. EJMM. 2015 July;24(3):21–6.

82. Ozdemir A, Elgormus Y. Diagnostic Value of Presepsin in Detection of Early-Onset Neonatal Sepsis. Amer J Perinatol. 2016 Nov 8;34(06):550–6.

83. Panda SK, Nayak MK, Rath S, Das P. The Utility of the Neutrophil-Lymphocyte Ratio as an Early Diagnostic Marker in Neonatal Sepsis. Cureus [Internet]. 2021 Jan 24 [cited 2025 Oct 21]; Available from: https://www.cureus.com/articles/50436-the-utility-of-the-neutrophil-lymphocyte-ratio-as-an-early-diagnostic-marker-in-neonatal-sepsis

84. Park IH, Lee SH, Yu ST, Oh YK. Serum procalcitonin as a diagnostic marker of neonatal sepsis. Korean J Pediatr. 2014;57(10):451.

85. Poggi C, Bianconi T, Gozzini E, Generoso M, Dani C. Presepsin for the Detection of Late-Onset Sepsis in Preterm Newborns. Pediatrics. 2015 Jan 1;135(1):68–75.

86. Pospisilova I, Brodska HL, Bloomfield M, Borecka K, Janota J. Evaluation of presepsin as a diagnostic tool in newborns with risk of early-onset neonatal sepsis. Front Pediatr. 2023 Jan 9;10:1019825.

87. Prashant A, Vishwanath P, Kulkarni P, Sathya Narayana P, Gowdara V, Nataraj SM, et al. Comparative Assessment of Cytokines and Other Inflammatory Markers for the Early Diagnosis of Neonatal Sepsis–A Case Control Study. Schulz C, editor. PLoS ONE. 2013 July 15;8(7):e68426.

88. Pravin Charles M, Kalaivani R, Venkatesh S, Kali A, Seetha K. Evaluation of procalcitonin as a diagnostic marker in neonatal sepsis. Indian J Pathol Microbiol. 2018;61(1):81.

89. Puello-Ávila AC, Cataño-Villegas AE. Utilidad de la proteína C-reactiva en la sepsis neonatal temprana. Rev chil infectol. 2021 Apr;38(2):169–77.

90. Rao L, Song Z, Yu X, Tu Q, He Y, Luo Y, et al. Progranulin as a novel biomarker in diagnosis of early-onset neonatal sepsis. Cytokine. 2020 Apr;128:155000.

91. Rashwan NI, Hassan MH, Mohey El-Deen ZM, Ahmed AEA. Validity of biomarkers in screening for neonatal sepsis – A single center –hospital based study. Pediatrics & Neonatology. 2019 Apr;60(2):149–55.

92. Rass AA, Talat MA, Arafa MA, El-Saadany HF, Amin EK, Abdelsalam MM, et al. The Role of Pancreatic Stone Protein in Diagnosis of Early Onset Neonatal Sepsis. BioMed Research International. 2016;2016:1–8.

93. Raynor LL, Saucerman JJ, Akinola MO, Lake DE, Moorman JR, Fairchild KD. Cytokine screening identifies NICU patients with Gram-negative bacteremia. Pediatr Res. 2012 Mar;71(3):261–6.

94. Rohsiswatmo R, Azharry M, Sari TT, Bahasoan Y, Wulandari D. TLR2 and TLR4 expressions in late-onset neonatal sepsis: Is it a potential novel biomarker? NPM. 2021 Sept 13;14(3):361–7.

95. Rosenfeld CR, Shafer G, Scheid LM, Brown LS. Screening and Serial Neutrophil Counts Do Not Contribute to the Recognition or Diagnosis of Late-Onset Neonatal Sepsis. The Journal of Pediatrics. 2019 Feb;205:105-111.e2.

96. Saldir M, Tunc T, Cekmez F, Cetinkaya M, Kalayci T, Fidanci K, et al. Endocan and Soluble Triggering Receptor Expressed on Myeloid Cells-1 as Novel Markers for Neonatal Sepsis. Pediatrics & Neonatology. 2015 Dec;56(6):415–21.

97. Schlapbach LJ, Graf R, Woerner A, Fontana M, Zimmermann-Baer U, Glauser D, et al. Pancreatic stone protein as a novel marker for neonatal sepsis. Intensive Care Med. 2013 Apr;39(4):754–63.

98. Sethi K, Verma RK, Yadav RK, Singh DP, Singh S. A study on bacteriological profile in suspected cases of neonatal sepsis and its correlation with various biomarkers in the rural population of a university hospital. Journal of Family Medicine and Primary Care. 2023 Oct;12(10):2313–7.

99. Shi J, Lu ZQ, Lin QM, Zeng W, Gu PJ, Yu Q, et al. The Role of Albumin in the Diagnosis of Neonatal Sepsis Over the Last 11 Years: A Retrospective Study. JIR. 2023 July;Volume 16:2855–63.

100. Sorsa A. Diagnostic Significance of White Blood Cell Count and C-Reactive Protein in Neonatal Sepsis; Asella Referral Hospital, South East Ethiopia. TOMICROJ. 2018 June 29;12(1):209–17.

101. Streimish I, Bizzarro M, Northrup V, Wang C, Renna S, Koval N, et al. Neutrophil CD64 as a Diagnostic Marker in Neonatal Sepsis. Pediatric Infectious Disease Journal. 2012 July;31(7):777–81.

102. Sumitro KR, Utomo MT, Widodo ADW. Neutrophil-to-Lymphocyte Ratio as an Alternative Marker of Neonatal Sepsis in Developing Countries. Oman Med J. 2021 Jan 15;36(1):e214–e214.

103. Tang YH, Jeng MJ, Wang HH, Tsao PC, Chen WY, Lee YS. Risk factors and predictive markers for early and late-onset neonatal bacteremic sepsis in preterm and term infants. Journal of the Chinese Medical Association. 2022 Apr;85(4):507–13.

104. Topcuoglu S, Arslanbuga C, Gursoy T, Aktas A, Karatekin G, Uluhan R, et al. Role of presepsin in the diagnosis of late-onset neonatal sepsis in preterm infants. The Journal of Maternal-Fetal & Neonatal Medicine. 2015 July 2;1–6.

105. Tosson AMS, Glaser K, Weinhage T, Foell D, Aboualam MS, Edris AA, et al. Evaluation of the S100 protein A12 as a biomarker of neonatal sepsis. The Journal of Maternal-Fetal & Neonatal Medicine. 2020 Aug 17;33(16):2768–74.

106. Tunc T, Cekmez F, Cetinkaya M, Kalayci T, Fidanci K, Saldir M, et al. Diagnostic value of elevated CXCR4 and CXCL12 in neonatal sepsis. The Journal of Maternal-Fetal & Neonatal Medicine. 2015 Feb 11;28(3):356–61.

107. Wilar R. Diagnostic value of eosinopenia and neutrophil to lymphocyte ratio on early onset neonatal sepsis. Korean J Pediatr. 2019 June 15;62(6):217–23.

108. Wu TW, Tabangin M, Kusano R, Ma Y, Ridsdale R, Akinbi H. The Utility of Serum Hepcidin as a Biomarker for Late-Onset Neonatal Sepsis. The Journal of Pediatrics. 2013 Jan;162(1):67–71.

109. Xiao T, Chen LP, Liu H, Xie S, Luo Y, Wu DC. The Analysis of Etiology and Risk Factors for 192 Cases of Neonatal Sepsis. BioMed Research International. 2017;2017:1–6.

110. Xiao T, Chen LP, Zhang L hua, Lai FH, Zhang L, Qiu Q feng, et al. The clinical significance of sCD14-ST for blood biomarker in neonatal hematosepsis: A diagnostic accuracy study. Medicine. 2017 May;96(18):e6823.

111. Yang KD, He Y, Xiao S, Ai Q, Yu JL. Identification of progranulin as a novel diagnostic biomarker for early-onset sepsis in neonates. Eur J Clin Microbiol Infect Dis. 2020 Dec;39(12):2405–14.

112. Ye Q, Du L zhong, Shao WX, Shang S qiang. Utility of cytokines to predict neonatal sepsis. Pediatr Res. 2017 Apr;81(4):616–21.

113. Yin W, Fang C, Fan X, Chen Y. Albumin and C-reactive protein as diagnostic markers for neonatal sepsis: a retrospective study. J Int Med Res. 2024 Mar;52(3):03000605241238993.

Methodology

A rapid evidence synthesis was conducted to identify the performance of biomarkers for neonatal sepsis. PubMed was searched for studies published between 1 January 2012 and 30 April 2024. All titles and abstracts of 4,906 articles was screened by two independent reviewers (BG and ABI). Any discord was subsequently adjudicated. Reviews, meta-analysis, non-human studies, non-neonatal populations, and studies without reported sensitivity and specificity were excluded. Full-text review confirmed eligibility. For each study, we extracted study design, location, sample size, biomarker, reference standard, and sensitivity and specificity. In some studies, methodological details (e.g. cohort versus case–control design or exact sample sizes) were not always clearly reported. Where possible, these were clarified from the available information and supplemented by careful review of tables, figures, and supplementary materials. Where multiple estimates were reported for the same biomarker within a study, the estimate with the highest sensitivity or highest balanced accuracy were used. This approach was applied across studies and reflects the WHO target product profile requirements where the intended use of these tests is as high-sensitivity triage tools. Biomarkers evaluated in fewer than two studies were excluded from

synthesis. A total of 22 biomarkers or combinations of biomarkers were identified. Biomarkers assessed in three or more studies; pooled analyses were performed.
